# Effect of conditioning and 3-year aging on the bond strength and interfacial morphology of glass-ionomer cement bonded to dentin

**DOI:** 10.1016/j.jds.2022.01.004

**Published:** 2022-01-22

**Authors:** Ahmed Zubaer, Rime Shamme Akter, Al Azad Salahuddin, Rahman Mir Ayubur, Sano Hidehiko, Hoshika Shuhei

**Affiliations:** a Department of Conservative Dentistry and Endodontics, Mandy Dental College and Hospital, Dhaka, Bangladesh; b Department of Restorative Dentistry, Division of Oral Health Science, Hokkaido University, Graduate School of Dental Medicine, Sapporo, Japan

**Keywords:** Adhesion, Dentin, Glass-ionomer cement, Micro-tensile bond strength, Scanning electron microscopy

## Abstract

**Background/purpose:**

Glass-ionomer cement (GIC) is bioactive and independent. Bioactivity, which is a big trend in restorative dentistry. When they actively stimulate with microbiological species besides their primary function of restoring tooth structure then restorative materials should called “bioactive” materials. The purpose of this study was to determine the bond stability and the change in interfacial ultra-structure of a conventional glass-ionomer cement bonded to dentin, with and without pre-treatment using a polyalkenoic acid conditioner.

**Material and methods:**

The occlusal dentin surfaces of six teeth were ground flat. Glass-ionomer cement was bonded to the surfaces either with or without polyalkenoic acid conditioning. The teeth were sectioned into 1-mm^2^ stick-shaped specimens. The specimens obtained were randomly assigned to two groups with different periods of storage in water: 1-week and 3-year. The micro-tensile bond strength (μTBS) was determined for each storage time. Additional specimens were prepared for interfacial analysis by using Scanning Electron Microscopy (SEM); they were produced with or without prior polyalkenoic acid conditioning in the same way as in the μTBS test.

**Results:**

There was no significant difference in μTBS to conditioned dentin and non-conditioned dentin (p > 0.05). The failures appeared to be of a mixed nature, although aging caused more areas of cohesive than adhesive failure in both groups.

**Conclusion:**

Aging did not reduce the bond strength of the conventional glass-ionomer cement to dentin with or without the use of a polyalkenoic acid conditioner. Remineralized dentin layer were observed in both conditioned and unconditioned 3-years specimens.

## Introduction

Nowadays we can carry out the tooth adhesion to enamel and dentin to an advance level by means of dental restorative materials such as glass-ionomer cement (GIC) and resin-based composites. GIC are bioactive and independent. Bio-compatibility or bioactivity, which is now a big trend in restorative dentistry.^1^ When they actively stimulate or direct tissue responses and they can control interactions with microbiological species besides their primary function of restoring or replacing missing tooth structure then dental restorative materials should be called “bioactive” materials.^2^ Remineralization and anti-microbial properties are the two major aspects of bioactivity. Those materials which are bioactive containing calcium silicate,^3^^,^^4^ calcium phosphate,^5^ hydroxyapatite^6^^,^^7^ etc, were reported to have remineralization ability. Regarding the anti-microbial property, the release of compounds with antibiotic-like efficacy were used to inhibit oral bacteria and biofilm.^8^^,^^9^

GIC is one of a dental bioactive material.^10^^,^^11^ It has a pH-buffering capacity, as it releases fluoride proportionally to the acidity.^12^^,^^13^ It has also both remineralization and anti-microbial ability^14^, ^15^, ^16^, ^17^ and it has been commonly used in the Atraumatic Restorative Treatment (ART) technique in developing countries for a long time.^18^ GIC can bond chemically to hydroxyapatite (HAp) and does not require light curing. Although it has a less demanding technique than resin-based restorations like resin composite, but often used clinically because of operator friendly technique, cost effectiveness and adhere directly to dental hard tissues even in a moist environment. It has a major advantage over resin composite that it has no conversion shrinkage and still an effective material in the case of deep cavities.^19^^,^^20^

GICs achieve such clinically suitable results and lowest annual failure rate in vivo even the bond strength of GIC may be much weaker compared with resin-based materials.^21^ Some studies have reported certain GICs adhere to tooth structure without pre-treatment^22^^,^^23^ but some other studies have reported by using surface pre-treatment the adhesion of GICs over dentin is improving.^24^^,^^25^ Previously studied the comparison between 1-week and 1-year of aging of GIC. This study is the continuation by using 1-week and 3-year aging of GIC bonded to dentin after getting result of 1-week and 1-year study.^26^

The purpose of this study was to assess the adhesion of the GIC-dentin by means of bond strength and interfacial morphology after 1-week and 3-year of aging, with and without surface pre-treatment. The null hypothesis tested in this study was that pre-treatment of dentin using a polyalkenoic acid conditioner did not affect the long-term durability of a conventional GIC.

## Materials and methods

### Microtensile bond strength test (μTBS)

The bond strength to dentin was determined using a standard micro-tensile bond strength test.^27^ The materials used in this study are shown in Table 1. Cavity conditioner (GC, Tokyo, Japan) and Fuji IX GP Extra (GC, Tokyo, Japan) which were used in this study. Six human molars, stored in a 0.5% chloramine T solution, were used within 1 month of extraction. The protocol of this research was approved by the Commission for Medical Ethics of Hokkaido University. The extracted molars were sectioned at the mid-coronal portion to create a flat dentin surface by using a low-speed diamond saw (Isomet 1000, Buehler, Lake Bluff, IL, USA). A standard smear layer was produced using #600 grit silicon carbide paper. The teeth were randomly divided into two groups of three teeth each. Prior to the application of the GIC, the dentin surface of the specimens in one group was pre-treated with a polyalkenoic acid conditioner (Cavity Conditioner, GC, Tokyo, Japan). This contains 3% Aluminum chloride as well as 20% polyalkenoic acid. The specimens in the other group did not receive any pre-treatment. The dentin surface was subsequently built up free-hand and in bulk with a conventional GIC (Fuji IX GP Extra, GC, Tokyo, Japan) to a height of 5–6 mm.

**Table 1:** The materials used in this study.

Product name	Composition
Cavity conditioner (GC, Tokyo, Japan)	20% Polyacrylic acid, Distilled water, Aluminum chloride hydrate, Food additive Blue No. 1
Fuji IX GP Extra (GC)	Polyacrylic acid, Aluminosilicate glass, Proprietary ingredient

After 1-week of storage in distilled water at 37 °C, the specimens were sectioned perpendicular to the bonding surface, to obtain 1-mm^2^ stick-shaped micro-specimens using an Isomet saw. The specimens were then randomly assigned to four groups (10 specimens each) according to age/storage time: 1-week and 3-year, i.e. the 1-week specimens were tested after sectioning while the rest continued in storage to 3-year. An absolute 3 teeth per experimental group with appropriate consideration of tooth dependency are required if the specimen is used as the statistical unit.^28^ At the relevant period, the micro-specimens were fixed to a jig with cyanoacrylate glue (Model Repair II Blue, Dentsply-Sankin, Ohtawara, Japan) and stressed in a testing device (EZ-test, Shimadzu, Kyoto, Japan) at a crosshead speed of 1 mm/min until failure occurred. The μTBS was calculated in MPa, derived by dividing the force applied (in N) at the time of fracture by the bonded area (in mm^2^). Statistical analysis was performed using one-way ANOVA (*α* = 0.05) and *post hoc* Tukey–Kramer multiple comparisons tests. The mode of failure was determined by examining the fractured surface at a magnification of × 80 using a stereo-microscope (Wild M5A, Heerbrugg, Switzerland).

### Scanning electron microscopy (SEM) interface analysis

Additional GIC specimens were prepared for examination using SEM (S-4000, HITACHI, Tokyo, Japan). For this, a further four teeth were randomly divided into two groups of two teeth each; the dentin was pre-treated with polyalkenoic acid conditioner in one group but not in the other. The procedure of bonding the GIC to dentin was the same as previously described in the μTBS test, before storage in distilled water for 1-week and 3-year at 37 °C. The GIC-bonded dentin specimens were sectioned perpendicular to the GIC/dentin interface using an Isomet diamond saw. From each tooth, seven or eight rectangular sections, of approximately 1 mm thickness each, were obtained. After storage for each time period, SEM sample preparation was performed in accordance with common procedures following a protocol described by Saikaew et al.^29^ Specimen were dried for 24 h. They were then fixed on aluminum stubs and coated with Pt–Pd alloy (E-1030, HITACHI, Tokyo, Japan) for 150 s. The GIC/dentin interface in each section was observed by SEM (S-4000, HITACHI, Tokyo, Japan) at an accelerating voltage of 10 kV. First, all the surfaces were examined at lower magnification (×80). Special features were further observed at ×800 and ×2000 magnifications.

## Results

### Microtensile bond strength (μTBS)

**Figure 1:**
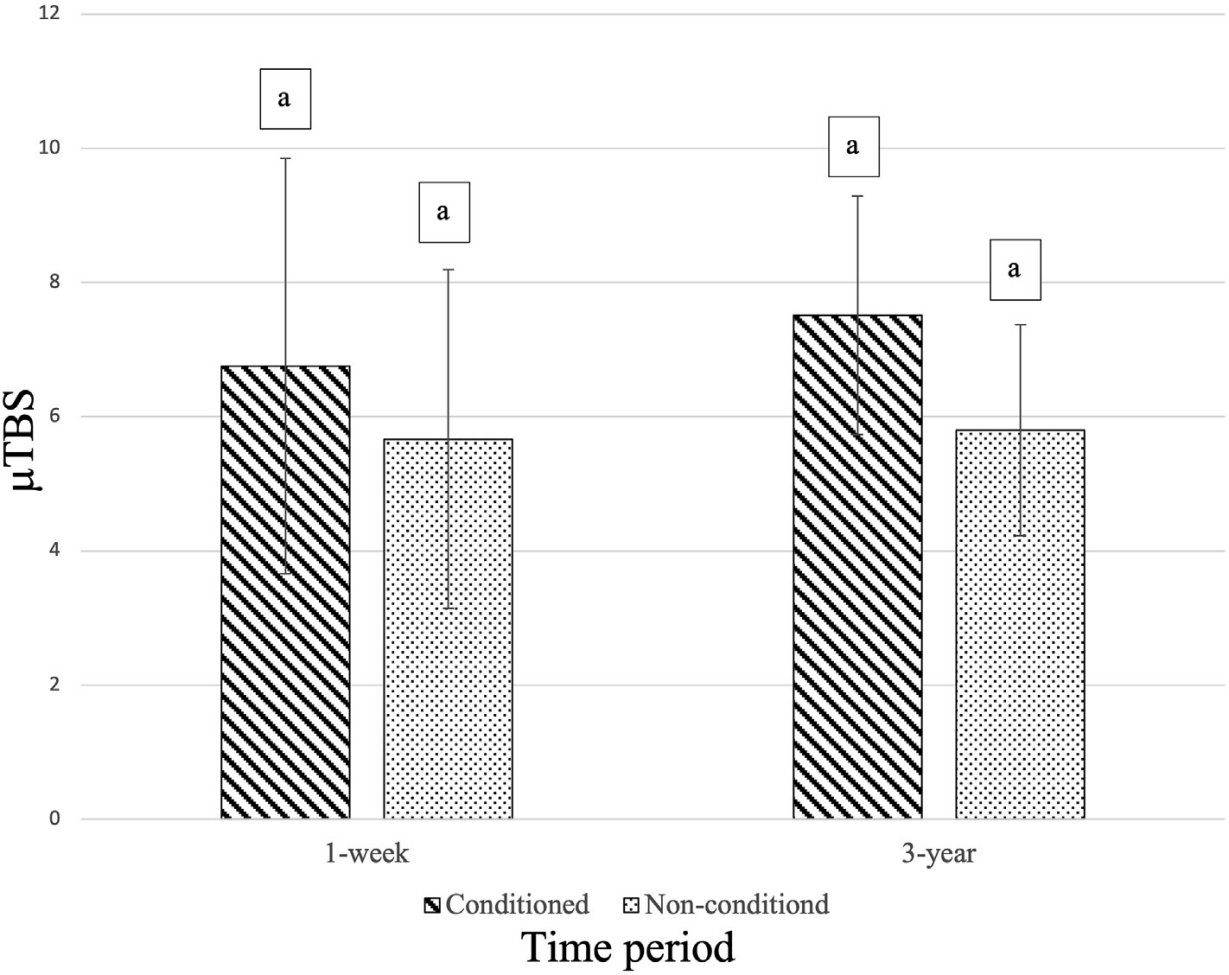
Micro-tensile bond strength of GIC bonded to polyalkenoic acid conditioned (Cavity Conditioner) and non-conditioned dentin for 1 week and 3 year. Mean μTBS are presented in MPa. n = 10. The same letters indicate no statistically significant difference (p > 0.05).

The mean μTBSs are presented in Fig. 1. No pre-testing failures (ptfs) were found in this study. There was no significant difference in μTBS when Cavity Conditioner was used at each period (p > 0.05). In addition, 3-year water storage did not show significant difference between conditioned and non-conditioned dentin in terms of μTBS results.

### SEM failure analysis

At 1-week, the failure patterns were generally of a ‘mixed’ nature, involving areas that failed at the interface and areas that failed cohesively within the GIC, for both the conditioned and non-conditioned groups. At 3-year, while the failure was still of a mixed nature, there was a tendency for more areas of cohesive failure. It appeared that aging of both conditioned and non-conditioned specimens caused them to fail slightly more frequently cohesively within the GIC.

### SEM interface analysis

**Figure 2:**
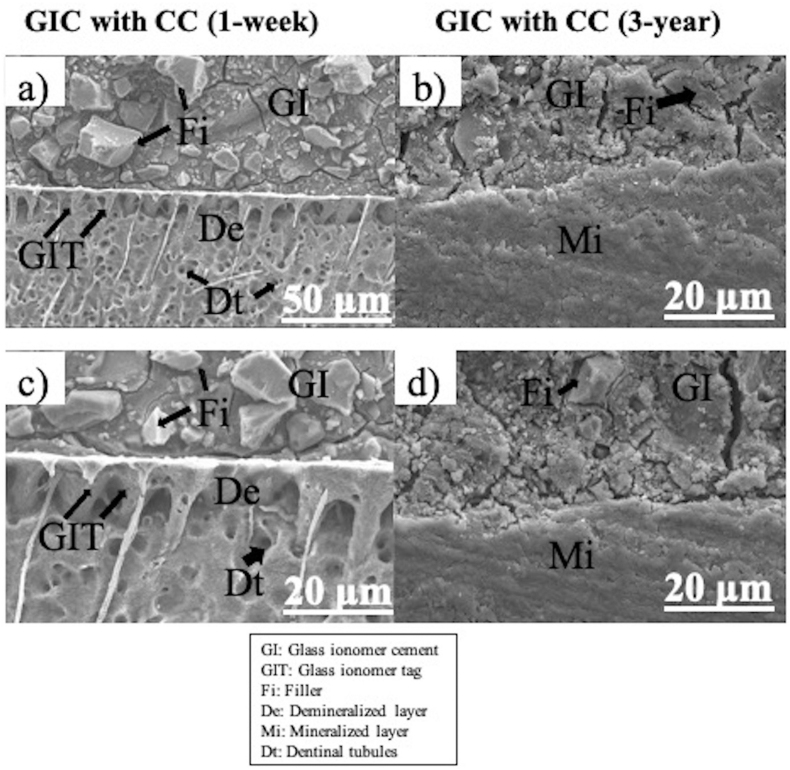
Representative SEM photomicrographs of the GIC/dentin interface with polyalkenoic acid conditioning using Cavity Conditioner stored for 1-week and 3-year (a,b,c,d). a = ×800 and b, c, d = ×2000. A partially demineralized dentin layer was formed on 1-week stored samples (Fig. 2a and c) whereas remineralized dentin layer was formed on 3-year samples (Fig. 2b and d). On 1-week, dentinal tubules were visible (Fig. 2a and c) while on 3-year there were no dentinal tubules seen (Fig. 2b and d). The GIC surface area were drier and several cracks were visible in long term stored samples (Fig. 2b and d). [GI = Glass ionomer cement; GIT = Glass ionomer tag; Fi = Filler; De = Demineralized Layer; Mi = Mineralized layer, Dt = Dentinal tubules].

**Figure 3:**
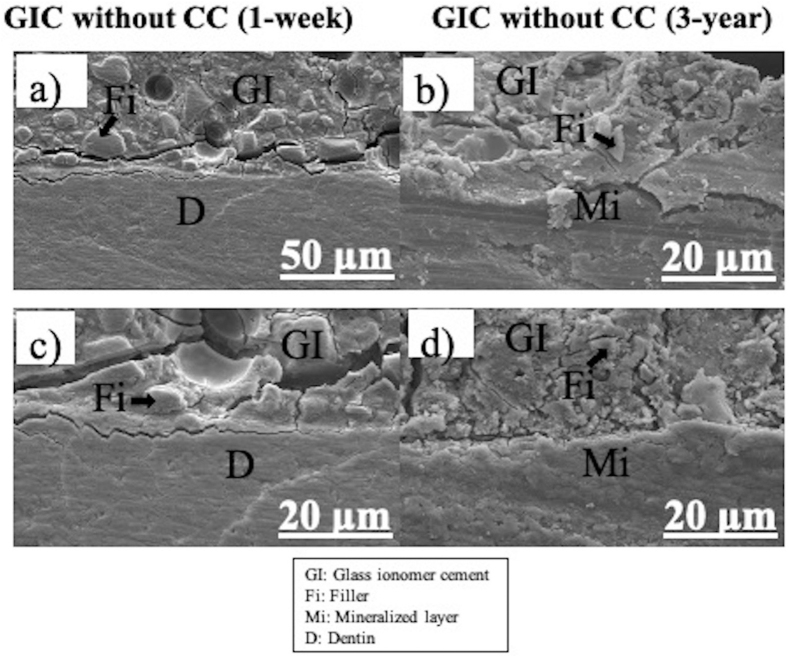
Representative SEM photomicrographs of the GIC/dentin interface without polyalkenoic acid conditioning stored for 1-week and 3-year (a, b, c, d). a, b, d = ×2000 and c = ×800. Observed intact-like dentin on 1-week and Mineralized dentin layer was observed on 3-year, but no dentinal tubules were seen on both 1-week and 3-year samples. Therefore, it is difficult to distinguish intact-like dentin and remineralized dentin on 3-year samples. However, on GIC surface area, several fillers were observed on both 1-week and 3-year storage time. [GI = Glass ionomer cement; Fi = Filler; Mi: Mineralized layer, D: Dentin].

Representative SEM images of the GIC/dentin interface with polyalkenoic acid conditioning stored for 1-week and 3-year are shown in Fig. 2a-d, while GIC/dentin with non-conditioned interface for 1-week and 3-year are shown in Fig. 3a-d.

With polyalkenoic acid conditioning, a shallow demineralized dentin layer was seen at the dentin-conditioned interface in 1-week (Fig. 2a and c). However, remineralized dentin layer was seen on 3-year image (Fig. 2b and d). Additionally, on GIC surface area, glass-ionomer tags were seen on 1-week specimen's image (Fig. 2a and c) whereas glass-ionomer tags were not visible on 3-year specimen's image (Fig. 2b and d). On 1-week, dentinal tubules were visible (Fig. 2a and c) while on 3-year there were no dentinal tubules seen (Fig. 2b and d). The GIC surface area were drier and several cracks were visible in long term stored samples (Fig. 2b and d). The conditioned interface has high viscous, so that the layer of interface is prominent. After interface observing, there were no sign of interface degradation in 3-year samples.

Representative SEM images of the GIC/dentin interface without polyalkenoic acid conditioning stored for 1-week and 3-year are shown in Fig. 3a-d. The GIC was closely attached to the dentin surface without any intervening layers detected. However, remineralized dentin layer might been seen on 3-year image Fig. 3 (b,d). No dentinal tubules were seen on both 1-week and 3-year samples. It is difficult to distinguish intact-like dentin and remineralized dentin on 3-year samples. The bond appeared intact. No clear signs of bond degradation were observed after 3-year of water storage.

## Discussion

The clinical ability of dental materials can be envisaged at laboratory settings by using μTBS test, especially after subjecting the specimens to aging challenges.^30^^,^^31^ The oral cavity has a light-hearted, various and heterogenous microbial or bacterial community in saliva. These bacteria inhabit the different surfaces of oral cavity and have advanced mechanisms to perception their environment. The microorganisms have some effect such as modulating systemic immune function, effect of local immunity, engaging in metabolism of substrate etc.^32^ In this study distilled water was used instead of saliva cause as the continuation of the previous study.^26^

In this study, by using μTBS test and SEM, the interfacial ultrastructure of GIC/dentin bonds and the long-term durability was studied respectively. From the μTBS test, there was no significantly difference between immediate and after 3 years of aging and the use of cavity conditioner did not make a significant difference to the μTBS (p > 0.05). As cohesive failure within the GIC tends to occur over time, this may be the reason why there was no significant difference in μTBS. The circumstances is that there was no significant difference in μTBS even when polyalkenoic acid conditioning was used. Although polyalkenoic acid is still recommended to maximize the ionic reaction with GIC, and to form insoluble calcium salts which facilitates wetting of the surface and increases the contact area.^33^, ^34^, ^35^, ^36^

In recent years, compared to resin-based adhesive interfaces, the interaction of GIC with dentin has been less commonly verified by high-resolution microscopy.^35^ SEM analysis in this study revealed that the interaction of GIC with dentin following some distinct patterns. After bonded with dentin by using polyalkenoic acid, a partially demineralized dentin layer was formed on 1-week stored samples (Fig. 2a and c) whereas remineralized dentin layer was formed on 3-year samples (Fig. 2b and d). On 1-week, dentinal tubules were visible (Fig. 2a and c) while on 3-year there were no dentinal tubules seen because of the remineralization effect (Fig. 2b and d). The GIC surface area were dried and several cracks were visible in long term stored samples (Fig. 2b and d) presumably because GIC has fragile characteristics. The conditioned interface has high viscous, so that the layers of interface is prominent. If we compare with 1-week and 3-year samples, the interface layer of 1-week was more prominent and visible than 3-year. This fact may be attributed due to the maturing effect of GIC, especially as when using polyalkenoic acid conditioning, the reaction of calcium and phosphate ions with GIC was stimulated and the remineralizing effect may have been supported as well with age. After interface observing, there were no sign of interface degradation in both 1-week and 3-year samples which was same by the previously reported researchers.^37^^,^^38^

When the GIC was applied without polyalkenoic acid conditioning, dentin demineralization was not observed (Fig. 3a–d), Just observed intact-like dentin on 1-week and presumably mineralized dentin layer on 3-year. No dentinal tubules were seen on both 1-week and 3-year samples. Whether with or without cavity conditioner, remineralized layer was seen after 3-year which is remarkable. In addition, according to previous study chemical bonding may occur.^39^ This was also demonstrated from the μTBS results, when GIC applied without prior polyalkenoic acid conditioning did not able to reveal significantly different bond strength in comparison with the conditioned dentin even though the limited micromechanical interlocking at up to 3-year of aging. In Fig. 3a, b of un-conditioned specimens, the dentin zone and GIC zone can see without any prime different types of layer and in μTBS, there were no significant difference between with and without the polyalkenoic acid conditioned group. An ultra-thin demineralized layer at the interface might exist.^26^

By using conditioners many researchers have shown an increase in the bond strength of GIC to dentin and a decrease in the amount of microleakage.^23^^,^^40^, ^41^, ^42^ This could be due to the removal of smear layer, elimination of debris, partial demineralization and formation of microporosities in the enamel and dentinal surfaces, enamel rod exposure, which results in an increased surface for microchemical and chemical bonding.^40^^,^^41^ Some researchers believe that there is no benefit in applying conditioners because the acidic nature of glass ionomer causes partial dissolution of the smear layer. The conflicting results reported in different studies can be the cause of residual dentin's thickness.^22^^,^^23^^,^^43^ GIC was applied with polyalkenoic acid conditioning might be clinically recommended for caries affected dentin, treated with deep dentin and old-aged patient.

Some studies have shown that GIC stored in saliva enhanced surface characteristics comparison with GIC stored in distilled water.^44^, ^45^, ^46^, ^47^ From saliva GIC may absorb some inorganic ions and this may improve the surface hardness over time. Further investigations should be conducted to assess the effect of GIC-dentin bond aging within saliva.

From the results of the μTBS test, pre-treatment of dentin using a polyalkenoic acid conditioner did not affect the long-term durability of a conventional GIC; hence, the null hypothesis should be accepted.

Further research will be conducted to access the effect of GIC-dentin bond aging within saliva and to access the effect of GIC on caries-affected dentin using polyalkenoic acid.

Within the limitations of this *in vitro* study, Aging did not reduce the bond strength of the conventional GIC to dentin whether the surface was pre-treated with a polyalkenoic acid conditioner or not. Remineralized dentin layer was observed in both conditioned and un-conditioned on 3-year specimens.

## Declaration of competing interest

The authors have no conflicts of interest relevant to this article.
